# Convergent evolution of hemoglobin switching in jawed and jawless vertebrates

**DOI:** 10.1186/s12862-016-0597-0

**Published:** 2016-02-01

**Authors:** Kim Rohlfing, Friederike Stuhlmann, Margaret F. Docker, Thorsten Burmester

**Affiliations:** Institute of Zoology, University of Hamburg, Martin-Luther-King-Platz 3, D-20146 Hamburg, Germany; Department of Biological Sciences, University of Manitoba, 50 Sifton Road, Winnipeg, MB R3T 2N2 Canada

**Keywords:** Agnatha, Ammocoete, Gene family, Hemoglobin switching, Myoglobin, Ontogeny, Oxygen, Phylogeny

## Abstract

**Background:**

During development, humans and other jawed vertebrates (Gnathostomata) express distinct hemoglobin genes, resulting in different hemoglobin tetramers. Embryonic and fetal hemoglobin have higher oxygen affinities than the adult hemoglobin, sustaining the oxygen demand of the developing organism. Little is known about the expression of hemoglobins during development of jawless vertebrates (Agnatha).

**Results:**

We identified three hemoglobin switches in the life cycle of the sea lamprey. Three hemoglobin genes are specifically expressed in the embryo, four genes in the filter feeding larva (ammocoete), and nine genes correspond to the adult hemoglobin chains. During the development from the parasitic to the reproductive adult, the composition of hemoglobin changes again, with a massive increase of chain aHb1. A single hemoglobin chain is expressed constitutively in all stages. We further showed the differential expression of other globin genes: Myoglobin 1 is most highly expressed in the reproductive adult, myoglobin 2 expression peaks in the larva. Globin X1 is restricted to the embryo; globin X2 was only found in the reproductive adult. Cytoglobin is expressed at low levels throughout the life cycle.

**Conclusion:**

Because the hemoglobins of jawed and jawless vertebrates evolved independently from a common globin ancestor, hemoglobin switching must also have evolved convergently in these taxa. Notably, the ontogeny of sea lamprey hemoglobins essentially recapitulates their phylogeny, with the embryonic hemoglobins emerging first, followed by the evolution of larval and adult hemoglobins.

**Electronic supplementary material:**

The online version of this article (doi:10.1186/s12862-016-0597-0) contains supplementary material, which is available to authorized users.

## Background

Hemoglobin (Hb) is a respiratory protein that facilitates the transport of oxygen (O_2_) from the respiratory surfaces (usually the skin, gills or lungs) to the inner organs [1]. Hb is present in almost all vertebrates, except some icefish species [2]. It is member of the globin protein family that is characterized by a conserved fold that includes a heme prosthetic group, by which the proteins reversibly bind O_2_ [1, 3]. In addition to Hb, other types of globins are present in the jawed vertebrates (Gnathostomata): myoglobin (Mb) [4], neuroglobin (Ngb) [5], cytoglobin (Cygb) [6–8], globin E (GbE) [9], globin X (GbX) [10], globin Y (GbY) [11] and androglobin (Adgb) [12]. A variety of functions other than O_2_ supply have been associated with these globins, including detoxification of reactive oxygen and nitrogen species (ROS/RNS) or signaling (for review, see [13]).

The Hb of the jawed vertebrates is a hetero-tetramer that is composed of two α- and two β-chains. The interaction of the chains leads to cooperative O_2_ binding [3]. Further modulation of the O_2_ affinity according to the physiological requirements is brought about by the interaction with organic phosphates (ATP, GTP, 2,3-diphosphoglycerate), CO_2_, and protons (Bohr effect), or by changing temperatures. Multiple, paralogous *α*- and *β*-genes have originated in evolution by gene duplication and divergence. During ontogeny, the O_2_ demand changes and, consequently, in many vertebrates distinct Hb chains are expressed in certain developmental stages [11, 14, 15]. For example, humans possess six *Hb* genes (α, β, γ, δ, ε, and ζ) [1]. Their differential expression results in embryonic, fetal, and adult forms of hemoglobin tetramers [1, 16]. The embryonic Hb consists of two α or ζ chains, respectively, plus two ε chains; the fetal Hb is composed of two α and two γ chains, which change to the adult Hb form (2 × α, 2 × β) during the first year after birth [1]. Embryonic and fetal Hb have higher oxygen affinities than adult Hb, which is essential to overcome the placental barrier in mammals [17].

The lamprey harbors five distinct globins: Adgb, GbX and Cygb, and functionally analogous Hbs and Mbs that evolved convergently from a common globin ancestor [18]. Lampreys, along with hagfishes, constitute the cyclostomes, the sole survivors of a lineage that diverged from the ancestor to the jawed vertebrates more than 500 million years ago [19, 20]. Like its counterpart in the jawed vertebrates, the lamprey Mb (aMb) is preferentially expressed in the skeletal muscle and presumably supports O_2_ to this tissue. The agnathan Hb (aHb) is structurally distinct from the gnathostome Hb, although it carries out similar functions. aHb is a monomer in its oxygenated form and associates into homodimers or tetramers when deoxygenated [21, 22]. Like the gnathostome Hbs, aHbs display cooperative O_2_ binding and a pH-dependent regulation of O_2_ affinity [23]. In the sea lamprey *Petromyzon marinus*, four distinct chains have been identified on the protein level that are components of the adult aHb [24–27]. However, analysis of the *P. marinus* genome revealed at least 14 additional *aHb* genes plus two pseudogenes [18]. Four of these closely resemble the known adult chains and probably are recent gene duplicates that cannot be distinguished from the main chain on the protein level. The expression patterns of the other nine *aHbs* remain unclear, leading to the speculation that they represent globin chains expressed in early developmental stages [18].

Sea lampreys (*P. marinus*) spend most of their life as filter-feeding larvae (ammocoetes), burrowed in the sediments of freshwater rivers [28]. After a dramatic metamorphosis involving major modifications to the morphology, physiology and behavior of the animal [29], the adult anadromous lampreys migrate to the sea, where they have a free-swimming hematophagous lifestyle. At the completion of the feeding phase, lampreys become sexually mature and return to fresh water, undergoing an upstream migration prior to spawning and death.

Early electrophoretic studies reported a shift from larval to adult aHb proteins during metamorphosis in a number of lamprey species [30–35]. More recently, Lanfranchi et al. [36, 37] demonstrated differential expression of one larval and two adult *aHb* genes before and after metamorphosis in the Po (Lombardy) brook lamprey (*Lampetra zanandreai*). No further studies, however, have examined the expression of other globins during ontogenesis of lampreys, and little attention has been paid to other developmental stages (e.g., embryogenesis, sexual maturation). The presence of at least 18 *aHbs* genes in the genome of *P. marinus* suggests intricate developmental regulation of expression [18]. To trace the hemoglobin switch in lampreys, we employed an RNA-seq and a qRT-PCR approach and studied the changes of the mRNA levels of *aHbs* and the other globins during four developmental stages of *P. marinus*: embryo, larval, parasitic adult, and reproductive adult.

## Results

### Expression pattern of aHb during development

We quantified the mRNA levels of the *aHb* genes of the sea lamprey (*P. marinus*) in different developmental stages by RNA-seq and qRT-PCR. First, a selection of putative housekeeping genes was evaluated in RNA-seq datasets for quality control and possible normalization. While the data indicate the integrity of the RNA, none of the putative housekeeping genes showed constant expression levels. Therefore, for RNA-seq normalization was done relative to the maximum expression level of *aHb1* in the adult reproductive datasets (Table 1), which was set to 100 arbitrary units (AU). All other expression levels were calculated as RPKM (reads per kilobase of transcript per million reads) and were related to that value. In qRT-PCR analyses, we estimated the mRNA copy numbers.

**Table 1:** Expression levels of globin genes in different developmental stages of the sea lamprey

Gene	Embryo	Larva	Adult-parasitic	Adult- reproductive
aHb1	0.0000	0.0767	8.9347	100.00
aHb2a	0.0100	0.0153	0.6641	0.2205
aHb2b	0.0353	0.0154	20.7233	10.4043
aHb2c	0.0200	0.0000	0.6943	0.2069
aHb2d	0.0000	0.0000	0.0000	0.0000
aHb3	0.0000	0.0000	12.4060	7.9237
aHb5a	0.0150	0.0000	7.3651	1.1106
aHb5b	0.0050	0.0000	6.9953	1.1677
aHb5d	0.0000	0.0000	12.6550	11.0104
aHb6	3.1568	4.1841	0.0425	0.1838
aHb7	0.8887	3.4145	2.7205	12.6774
aHb8	0.0200	0.0000	2.5884	0.8084
aHb9	2.5544	0.1310	0.0000	0.0000
aHb10	2.0739	0.0000	0.0000	0.0052
aHb11	1.5650	0.0291	0.0000	0.0646
aHb12	0.1424	1.1575	0.0000	0.9181
aHb13	0.7416	4.1488	0.0074	0.0747
aHb14	1.1001	7.0349	0.0074	0.0721
aMb1	0.4822	4.8613	13.1111	45.9467
aMb2	0.0893	1.9698	0.2619	0.0996
Cygb	1.1232	0.2356	0.2934	0.1723
GbX1	0.0164	0.0000	0.0000	0.0000
GbX2	0.0000	0.0000	0.0000	0.0038

The values were derived from the RNA-seq data and related to the highest expression level of *aHb1*, which was set to 100 arbitrary units (AU)

**Fig. 1:**
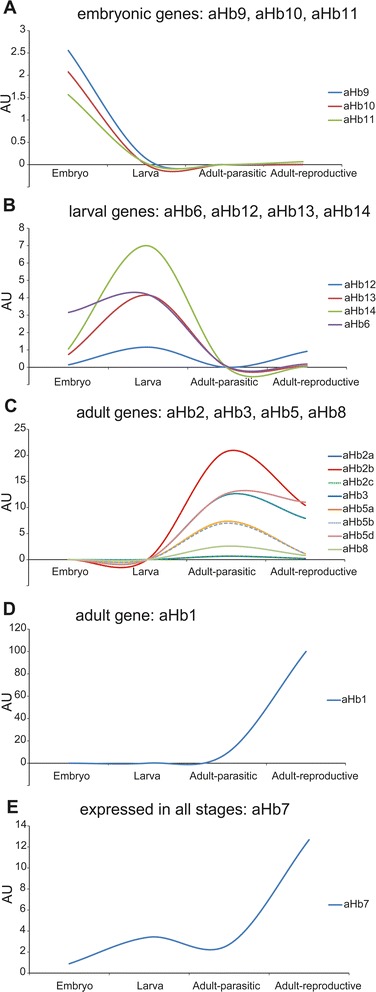
Expression profiles of sea lamprey *aHbs* quantified by RNA-seq. The *aHb* genes were displayed as predominantly expressed in the embryonic (**a**), larval (**b**), adult-parasitic (**c**) and adult-reproductive (**d**) stages. aHb7 (**e**) was not assigned to any specific developmental stage. The expression level is indicated as arbitrary units (AU), relative to the highest RPKM of the pool of adult-reproductive *aHb1* expression, which is set to 100. Note that the expression levels of aHb2a and aHb2c, and aHb5a and aHb5b, respectively, were almost identical

**Fig. 2:**
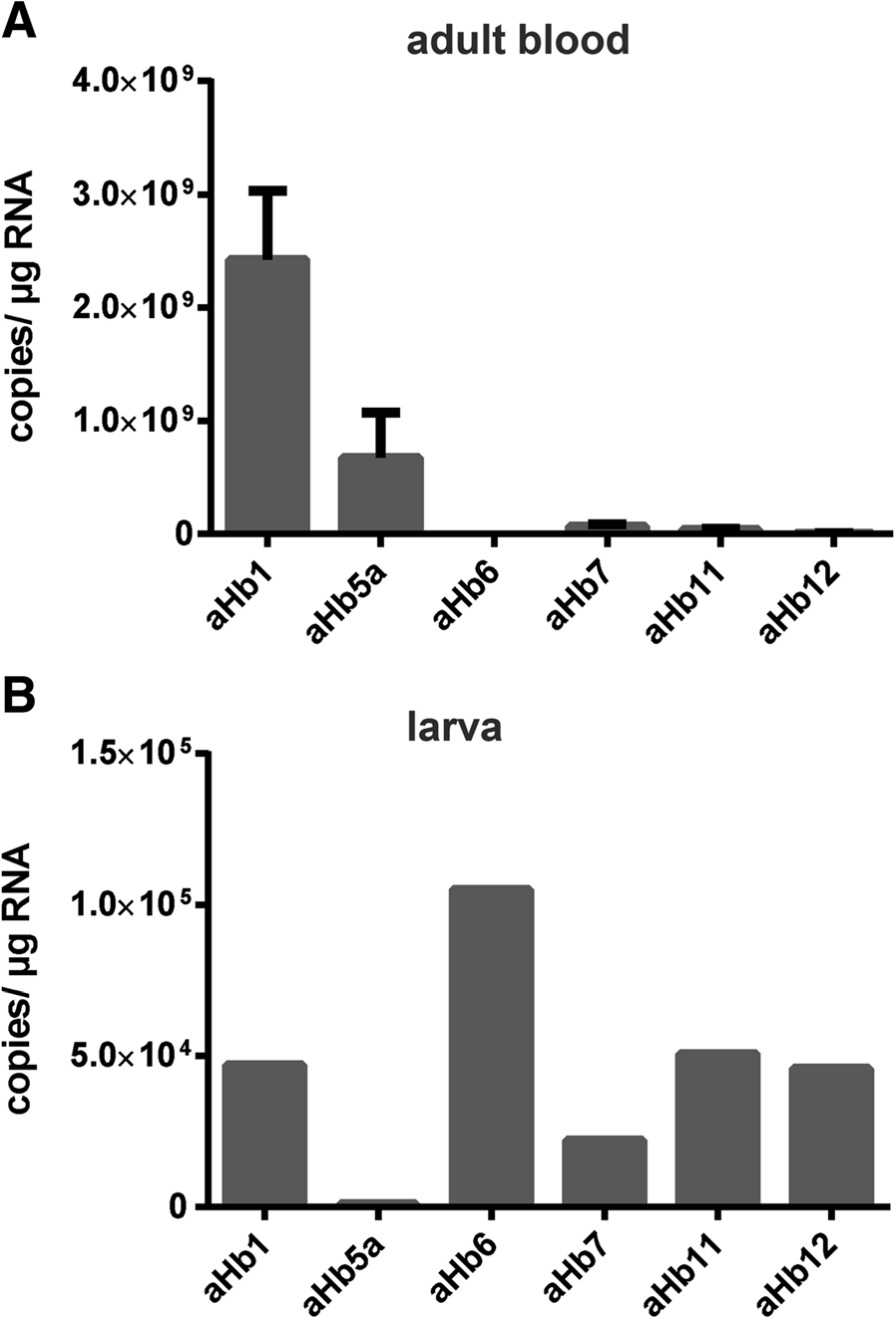
Quantification of mRNA levels of selected sea lamprey globins. Adult blood (**a**) or whole ammocoete (**b**) were used. The transcript abundance was quantified by qRT-PCR of *aHb1*, *aHb5a*, *aHb6*, *aHb7*, *aHb11*, *aHb12* (a, b)

Both methods indicated differential expression of the globins throughout development. Analysis by RNA-seq showed that *aHb9*, *aHb10*, and *aHb11* are almost exclusively expressed in the embryo (Fig. 1a; Table 1). Compared to the high levels of *aHb* in the adult stage, the overall expression levels in the total embryo were comparably low (AU < 3). *aHb6*, *aHb12*, *aHb13*, and *aHb14* are mainly expressed in the larval stage, with the highest levels for *aHb14* (7 AU) (Fig. 1b); notable amounts of *aHb6* were also detectable in the embryo (1.1 AU). The other NGS datasets were separated into an adult-parasitic stage and an adult-reproductive stage. The genes representing the known chains of *P. marinus aHb* [24–27], *aHb2a*, *b*, *c*, *aHb3*, *aHb5a*, *b*, *d* and *aHb8* are most highly expressed in the adult-parasitic stage (Fig. 1c). Notable amounts of *aHb2b*, *aHb5d* and *aHb3* are also detectable in the later adult stage. *aHb1* is the most highly expressed globin gene of *P. marinus*. While it is not expressed in the embryo and only traces could be found in the larva (AU = 0.076; Table 1), it is a component of the adult aHb and reaches 100 AU in the reproductive stage (Fig. 1d). This finding is supported by the qRT-PCR results employing adult blood (Fig. 2a). In the adult, notable amounts of mRNA were also detected for *aHb5*, while the other analyzed *aHbs* display low levels. The qRT-PCR further showed that *aHb6*, *aHb11* and *aHb12* are mainly expressed in the ammocoete (Fig. 2b). The pattern of *aHb7* differs from that of the other *aHb* genes, being expressed with increasing levels throughout ontogeny (AU = 0.88 to 12.7) (Figs. 1e; 2a, b; Table 1).

### Ontogeny of the tissue globins

**Fig. 3:**
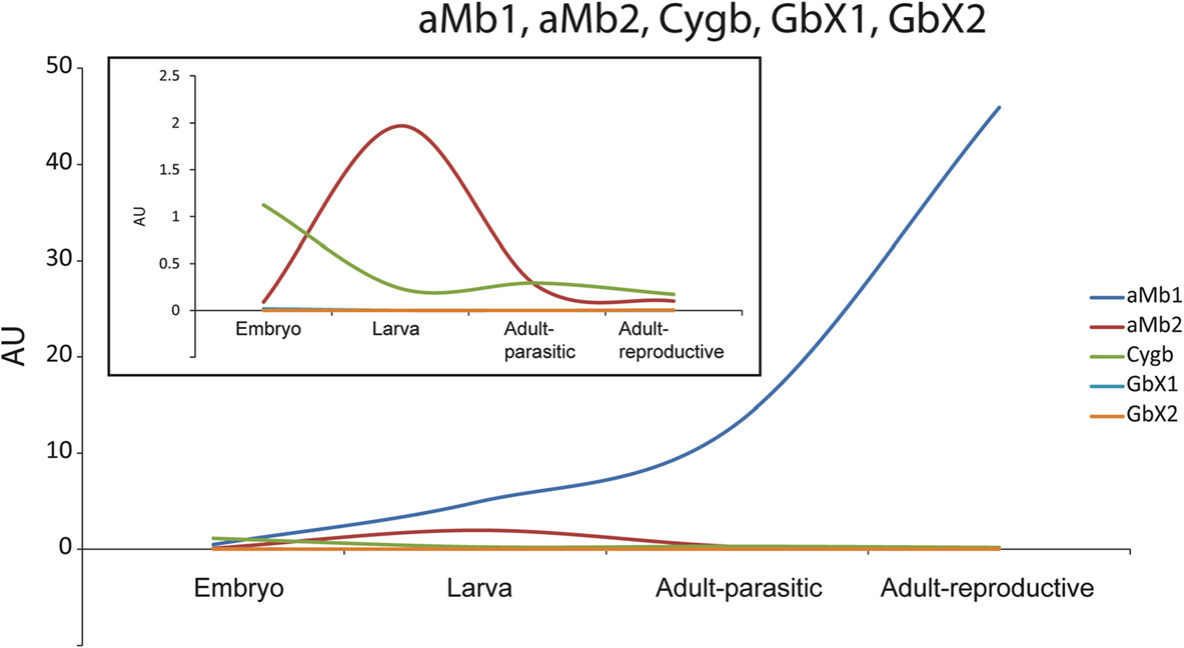
Expression profiles of sea lamprey globins quantified by RNA-seq. The expression level of *aMb1*, *aMb2*, *Cygb*, *GbX1* and *GbX2* are indicated as AU (see above). The mRNA levels of *aMb2*, *Cygb*, *GbX1*, *GbX2* were additionally displayed in the inset

In addition to the *aHbs*, which code for globin chains that transport O_2_ in the blood, the globin repertoire of the sea lamprey includes *Cygb*, *aMb1*, *aMb2*, *GbX1* and *GbX2*, which reside in the tissues [18]. We traced the expression changes of these genes by RNA-seq. Among them, the mRNA level of *aMb1* is the highest; while only 0.482 AU were found in the embryo, its expression constantly increases until it reaches 46 AU in the adult stage (Fig. 3; Table 1). The level of *aMb2* and *Cygb* are low throughout all developmental stages. The *aMb2* transcript is mainly expressed in the larva (AU = 1.97), and *Cygb* is highest in the embryo (AU = 1.12) (Fig. 3, inset). The two *GbX* transcripts showed a low but differential expression: while *GbX1* was found exclusively in the embryo (0.016 AU), *GbX2* is only expressed in the adult (0.0038 AU) (Fig. 3, inset; Table 1).

### Evolution of stage-specific hemoglobins in sea lamprey

**Fig. 4:**
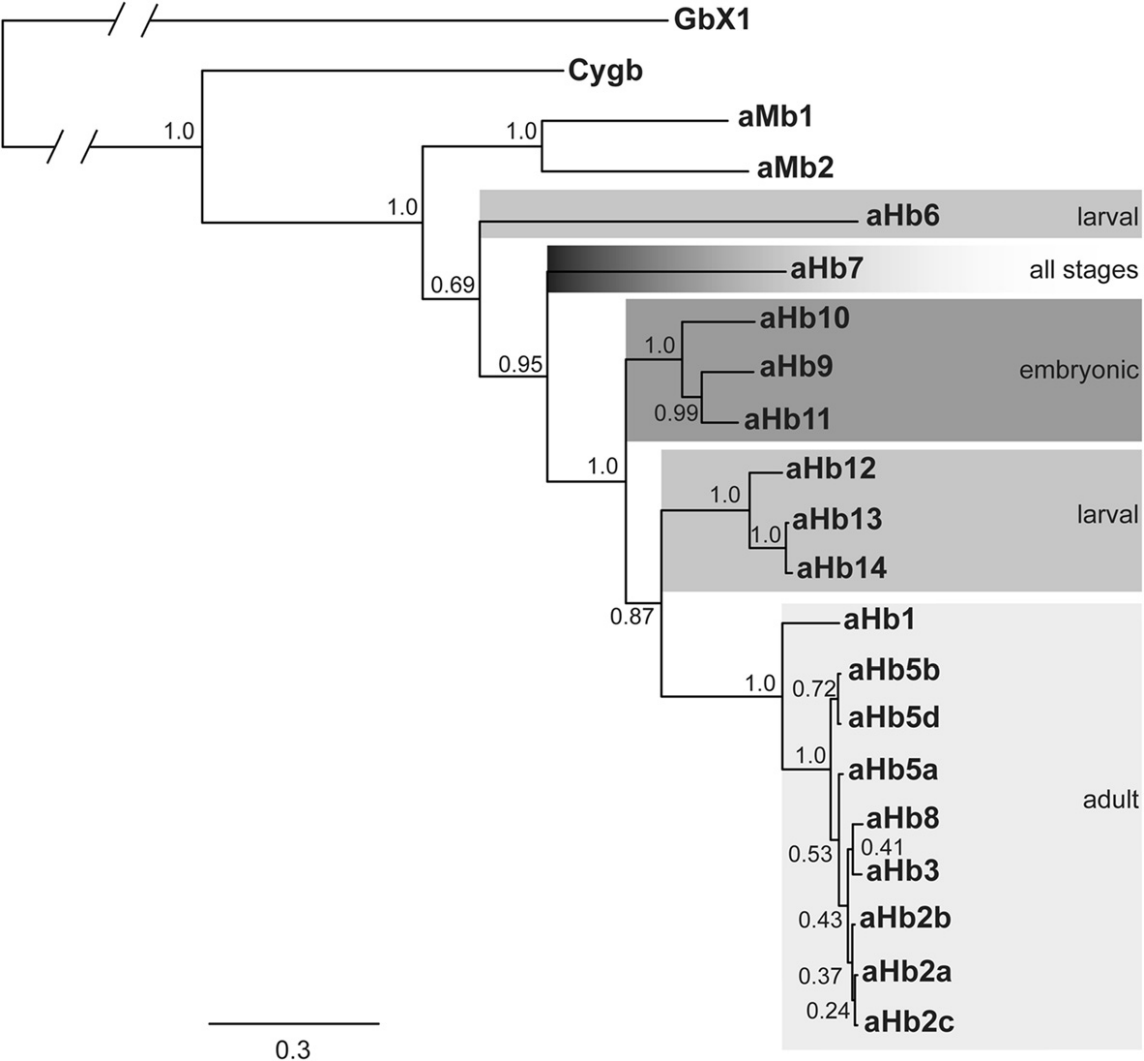
Mapping of the stage specific expression patterns onto a Bayesian phylogenetic tree of sea lamprey globins. The developmental stages are shaded in different grey scales. The numbers at the nodes are posterior probabilities. The bar represents 0.3 PAM distance

We mapped the expression patterns onto a Bayesian phylogenetic tree of the agnathan globins. For simplification, only globins of the sea lamprey were considered. The basic topology of the tree was similar to that retrieved in a previous analysis [18]. The aHbs build a single clade, which forms the sister group of the two aMbs (Fig. 4). In most cases, the aHbs that are predominantly expressed during a certain phase of the life cycle, cluster together in specific clades (Fig. 4). Only aHb6, which is mainly expressed in both the embryo and the larva, and aHb7, which is expressed throughout the lifecycle, do not match this pattern. Among the sea lamprey aHbs, aHb6 diverged first, followed by aHb7. Next comes a clade composed of the embryonic aHbs (aHb9-11), which forms the sister group of the larval aHbs (aHb12-14) and adult aHbs (aHb1-3, 5, 8).

## Discussion

### Changes in hemoglobin composition during the ontogeny of the sea lamprey

Protein studies identified four distinct aHb chains in adult sea lamprey [24–27], which correspond to *aHb1*- *aHb3* and *aHb5* [18]. In fact, the mRNAs of these *aHbs* (including the *aHb2* and *aHb3* variants that represent recent gene duplicates) are the most strongly expressed adult *aHbs. aHb8* represents an additional adult chain, which has been missed in previous protein sequencing studies, probably due to its similarity with *aHb3.* The composition of the aHb appears to change during the transition of the adult lamprey from the parasitic to the reproductive stage (Fig. 1). While the mRNA levels of *aHb2*, *3, 5*, and *8* slightly decrease, *aHb1* markedly rises to the highest levels measured for any globin in the sea lamprey. Thus, the composition of the aHb changes during adult life. In contrast, electrophoretic studies on the hemoglobin of the Austalian parasitic anadromous lamprey *Mordacia mordax* found no difference between small adults just prior to parasitic feeding and large adults returning on their spawning migration [34]. Thus, although it has long been known that a hemoglobin switch occurs in lampreys at metamorphosis, a further change in Hb composition at maturation has not been previously characterized. It remains uncertain which physiological constraints alter the O_2_ demands in the reproductive form of the sea lamprey requiring such a change in aHb composition.

While protein sequencing supports the interpretation of aHb1- aHb3, and aHb5 (and their variants) as the main components of the adult aHb, the nature of the other aHbs can only be inferred by tracing their stage-specific expression. The embryonic aHb probably consists of at least *aHb9*, *aHb10*, and *aHb11* encoded chains. Traces of mRNA of these *aHbs* can be also found in the adult reproductive (but not parasitic) stage, which may be interpreted as eggs that were included in the mRNA preparation. The larval aHb at least consists of chains encoded by *aHb12*, *aHb13*, and *aHb14* transcripts. This interpretation is further backed by the aHbA of the Po brook lamprey [37], which cluster in phylogenetic analyses with a clade formed by aHb13 and aHb14 of the sea lamprey [18]. The nature of *aHb6* and *aHb7* transcripts is less clear. aHb6 is most likely component of both the embryonic and the larval aHbs. The levels of *aHb7* do not show indications of a clear switch but rather constantly increase during development, which hints at a more specific role.

### Convergent evolution of hemoglobin switching in jawed and jawless vertebrates

The data demonstrated three hemoglobin switches in the sea lamprey, which can be considered as functionally analogous to the Hb switch of the jawed vertebrates [1, 38]. The first switch occurs from the embryonic to the larval stage. An analogous Hb switch during early development occur in fish [14, 15] and in amphibians [39]; it can also be considered analogous to the switch from embryonic to fetal Hb during mammalian development [1, 16]. The second switch takes place during metamorphosis from the larval to the adult lamprey. Again, analogous switches occur in the jawed vertebrates [11, 40–42]. A third, minor switch occurs during the transition of the parasitic to the reproductive adults (see above).

However, the *Hb* genes of jawed (Gnathostomata) and jawless (Agnatha) vertebrates are not homologous but emerged convergently [18, 43]. Thus, also the hemoglobin switch in these taxa must have emerged convergently and the scenario of an ancient common origin of the vertebrate hemoglobin switch can be excluded. The expression of different *Hb* genes that probably result in changes of O_2_ affinities may be instrumental to adapt to different O_2_ requirements of different developmental stages.

### Ontogeny recapitulates phylogeny of lamprey hemoglobins

The phylogenetic tree (Fig. 4) remarkably mirrors the ontogeny of *aHb* expression: embryonic aHbs diverged first, followed by larval and adult aHbs. Only aHb6 and aHb7 deviate from this pattern. aHb6 is an embryonic/larval aHb that forms the earliest branching clade among the agnathan aHbs; in the tree, it is followed by aHb7, which is expressed throughout the development of the sea lamprey, with increasing levels in later life. This tree topology may have emerged by chance; however, it is also conceivable that it reflects the different changes in lifestyles that have occurred during evolution. The embryonic aHbs may mirror the hemoglobin in the agnathan ancestor; the aHbs of the filter-feeding lamprey larva may reflect the aHbs that emerged with the first agnathans, which had a similar life style (for example, *Haikouichthys* and *Myllokunmingia*; [44, 45]. With the evolution of a free-living, hematophagous lamprey, the adult forms of hemoglobin may have emerged. This interpretation is supported by the fact that the differentially expressed aHbs diverged before the hagfish and lampreys diverged [18].

### Regulation of hemoglobin expressions

The expression of *Hb* in the Gnathostomata is controlled by sequential activation and repression of *Hb* genes that are chromosomally linked in clusters. In amphibians and Teleostei, the ancestral arrangement of the tandemly arranged *Hbα* and *Hbβ* is well preserved [11, 40, 46–49]. In the Amniota, the genes that code for *Hbα* and *Hbβ* are arranged on different chromosomes; this arrangement is the result of independent translocations of the *Hbβ* clusters [50, 51]. Currently, little is known about the arrangement of *aHb* genes in the lamprey [18] due to the fragmentary nature of the genome assemblies of *P. marinus* [52] and the Arctic lamprey *Lethenteron camtschaticum* [53]. However, the adult genes *aHb3*, *aHb5a*, *aHb5b*, *aHb5c*, and *aHb8* are tandemly arranged in the same orientation in a single cluster, which may be an indicator for a coupled regulation. The same applies for the larval genes *aHb12*, *aHb13* and *aHb14* [18].

### Switches in myoglobin and GbX expression in the sea lamprey

In addition to the *aHbs*, also *aMb* and *GbX* show differential expression throughout development. The sea lamprey possesses two *aMb* genes, which are – according to qRT-PCR studies – mainly expressed in the heart tissue, but were also found in muscle and brain [18]. *aMb1* mRNA levels constantly increase from the embryo to the adult stages, which is probably associated with an enhanced heart function that requires a better O_2_ supply. It remains uncertain why *aMb2* peaks in the larval stage. The expression patterns of the two *GbX* genes show a clear-cut difference, which restricts *GbX1* to the embryo and *GbX2* to the adult reproductive stage (Table 1). This pattern suggests that the divergence of *GbX1* and *GbX2* has led to a subfunctionalization of these genes, which probably have specific roles in each developmental stage.

## Conclusions

The expression of distinct genes during animal development allows for the emergence of specific structures and the adaptation to specific metabolic requirements. The developmentally controlled expression of Hbs is probably the most prominent example of a switch in gene expression during the ontogeny of humans and other jawed vertebrates [1, 11, 14–16]. Here we have shown that in the sea lamprey, three hemoglobin switches occur, accounting for specific sets of aHb chains in the embryo, the larva, the parasitic adult and the reproductive adult. Because Hbs evolved convergently in gnathostomes and agnathans [18], the Hb switching in jawed and jawless vertebrates must also have evolved convergently. Surprisingly, the ontogeny of sea lamprey aHbs recapitulates their phylogeny. This has not been observed for the gnathostome Hbs.

## Methods

### Data collection and gene expression analyses with RNA-seq data

Next-generation sequencing (NGS, *i.e.* 454 or Illumina technologies) reads from four different development stages of *P. marinus* were obtained from NCBI (http://www.ncbi.nlm.nih.gov/sra). A total of 22 datasets were used: seven from embryonic stages (Illumina), four from the larval stages (two 454, two Illumina), six of adult-parasitic stages (two 454, four Illumina) and five of adult stages (three LS 454, two Illumina). Information on the datasets, including developmental stages and tissue, respectively, are given in Table 2. The transcript abundance of all annotated sea lamprey globin genes [18] were estimated using the RNA-seq analysis tool of CLC Genomics Workbench. The globin cDNAs and putative housekeeping genes were used as reference sequences with adjusted mapping options due to the high similarity among the aHb sequences (mismatch cost: 2, insert cost: 3, deletion cost: 3, length fraction: 0.99, similarity fraction: 0.99) in a global alignment with the SRA datasets. Each RNA-Seq dataset (Table 2) was individually mapped against the reference sequences and normalized according to the transcript length and the size of the dataset. Because none of the tested putative housekeeping genes showed constant expression throughout ontogeny, the resulting RPKM values were used. Due to the small reference dataset, the highest aHb expression level (*aHb1* in the adult reproductive datasets) in RPKM was set to 100 arbitrary units (AU). All other RPKMs were related to that value.

**Table 2:** RNA-seq data sets used in expression analyses. For each given stage, the datasets were combined; the analyses of the individual samples are given in Additional file 3: A3

Developmental stage	Accession number	Tissue and specific stage	Sequencing method
Embryo	SRX110035	Neural Crest Migration, Stage 24c2	Illumina
	SRX110034	Neural Crest Migration, Stage 24c1	Illumina
	SRX110033	Neural Crest Migration, Stage 23	Illumina
	SRX110032	Neurula, Stage 22b	Illumina
	SRX110031	Neurula, Stage 22a	Illumina
	SRX110030	Gastrula, Stage 20	Illumina
	SRX110029	Late Blastula, Stage 18	Illumina
Larva	SRX109766	Liver	454
	SRX109765	Brain	454
	SRX110023	Kidney	Illumina
	SRX109770	Intestine	Illumina
Adult-parasitic	SRX110026	Distal intestine	Illumina
	SRX110025	Proximal intestine	Illumina
	SRX110024	Kidney	Illumina
	SRX109767	Liver	454
	SRX109769	Liver	Illumina
	SRX109761	Olfactory epithelium	454
Adult-reproductive	SRX109768	Brain	Illumina
	SRX109764	Brain	454
	SRX109762	Olfactory epithelium	454
	SRX110028	Kidney	Illumina
	SRX110027	Intestine	Illumina

### Animals

Two adult sea lampreys (63 cm, 731.1 g and 58 cm, 535.3 g) were collected from the Elbe estuary in June 2013 with the permission of the *Niedersächsisches Landesamt für Verbraucherschutz und Lebensmittelsicherheit*. Animals were handled according to regulations of the German Animal Welfare Act (*Tierschutzgesetz*). Samples of blood (each about 1 ml), heart and gonads were harvested, immediately placed on dry ice and stored at -80 °C. One larva (ammocoete) of the sea lamprey was provided by the U.S. Geological Survey, Hammond Bay Biological Station in Ray Road, Millersburg, Michigan. The larva (7.2 cm, 0.48 g) was collected in May 2014 from the Chippewa River, Canada. The collection, storage, and use of sea lamprey followed the guidelines of the U.S. Geological Survey. The animal was cut into pieces of 0.5 cm and immediately stored in RNAlater (Qiagen, Hilden, Germany), shipped at room temperature and stored subsequently at -20 °C.

### RNA extraction

Total RNA from each sample was extracted using peqGOLD Trifast (PEQLAB, Erlangen, Germany) and the Crystal RNA Mini Kit (BiolabProducts, Gödenstorf) according to manufacturer’s instructions. Two blood samples were thawed on ice and homogenized with a pestle in 750 μl peqGOLD Trifast. The heart, gonads and larval samples were rinsed in PBS (140 mM NaCl, 2.7 mM KCl, 8.1 mM Na_2_HPO_4_, 1.5 mM KH_2_PO_4_, pH = 7.1) that had been treated with diethylpyrocarbonate, ground in liquid nitrogen with mortar and pestle and homogenized in 1 ml peqGOLD Trifast. After the addition of one fifth volume of chloroform, the aqueous phase was purified using the silica column of the Crystal RNA Mini Kit with on-column DNase treatment (RNase-free DNase, Qiagen).

### Reverse transcription and cDNA cloning

The RevertAid H Minus First Strand cDNA Synthesis Kit (Thermo Fisher Scientific, Bonn) was used for reverse transcription. For cDNA cloning and quantitative real-time expression analyses, 1 μg and 750 ng, respectively, isolated RNA were reverse transcribed with oligo-(dT)_18_ primer in a total volume of 20 μl. Gene-specific oligonucleotides (Additional file 1: Table A1) were used for amplification of selected sea lamprey globin genes. The genes were chosen to cover each aHb branch of the phylogenetic tree [18], thus representing putative embryonic, larval and adult hemoglobins. The PCR products were cloned into standard cloning vectors (pGEM-T, Promega, or pJET1.2, Thermo Scientific) and sequenced by a commercial service (GATC, Konstanz, Germany).

### Quantitative real-time reverse transcription polymerase chain reaction (qRT-PCR)

The expression of selected globin mRNAs (*aHb1*, *aHb5a*, *aHb6*, *aHb7*, *aHb11*, *aHb12*, *Cygb*, *aMb1* and *aMb2*) was estimated by qRT-PCR on an ABI 7500 real-time PCR system. Amplification was performed using the ABI Power SYBR Green master mix (Applied Biosystems, Darmstadt, Germany) with 40 cycles (95 °C for 15 s, 60 °C for 15 s, 72 °C for 30 s, detection at last step), employing intron-spanning primers (Additional file 1: Table A1). A cDNA amount equivalent to 18.75 ng RNA was used per reaction; the experiments were carried out as triplicates. Negative controls without cDNA were run as single experiments. The specificity of the amplifications was controlled by dissociation curve analyses. The standard curve method with recombinant plasmids in tenfold serial dilution was used to calculate the total mRNA copy number. The samples were normalized according to 1 μg total RNA.

### Phylogenetic analyses of sea lamprey globins

The sea lamprey globin repertoire was extracted from a published dataset [18]. In the new dataset, all partial sequences and pseudogenes were excluded but closely related proteins with identical amino acid sequences were retained, thus a set of 21 sequences was used (GbX1, Cygb, two aMbs, 17 aHbs) (Additional file 2: Figure A1). The phylogenetic relationship among these globins was estimated using MrBayes on XSEDE 3.2.6 [54, 55] (https://www.phylo.org/) with the LG model of amino acid evolution. The multiple alignment of amino acid sequences was generated by MAFFT with L-INS-I strategy [56, 57]. Two independent runs with one cold and three heated chains were performed for 5x10^6^ iterations and trees were sampled every 1000th generation. Posterior probabilities were estimated on the final 3,000 trees. GbX1 were used as outgroup because of its divergence from the other globin lineages before the separation of Protostomia and Deuterostomia [10, 58].

### Availability of data and materials

All supporting data are available in the Additional file 1, Additional file 2 and Additional file 3.

## Additional files

Additional file 1: Table A1. Oligonucleotides used for RT-PCR and qRT-PCR. (PDF 26 kb) Additional file 2: Figure A1. Sequence alignment (FASTA format) of the globin sequences of P. marinus. (PDF 8 kb) Additional file 3:
**Analyses of individual sequence datasets.** (XLSX 51 kb)

## Abbreviations

Adgb: androglobin
aHb: agnathan hemoglobin
aMb: agnathan myoglobin
AU: arbitrary units
Cygb: cytoglobin
GbE: eye-specific globin
GbX: globin X
GbY: globin Y
Hb: hemoglobin
Mb: myoglobin
Ngb: neuroglobin
RPKM: reads per kilobase of transcript per million reads
