# Supplementary material for: Convergent evolution of hemoglobin switching in jawed and jawless vertebrates
Source: BMC Evol Biol. 2016 Feb 1;16:30. doi: 10.1186/s12862-016-0597-0 (PMC4736134; doi:10.1186/s12862-016-0597-0)
Supplement: Additional file 1: Table A1. — Oligonucleotides used for RT-PCR and qRT-PCR. (PDF 26 kb) [file 12862_2016_597_MOESM1_ESM.pdf]

Additional Table A1. Oligonucleotides used for RT-PCR and qRT-PCR

Gene	Label	5'-3'sequence
**aHb1**	Pma_aHb1_for	GACTCTGGAAGTGTCCCAG
	Pma_aHb1_rev	GCTCAGGTCCAGCTACTGA
	Pma_aHb1_qpcr_f	CAGCACCTTGAAGTACTGGGGGTC
	Pma_aHb1_qpcr_r	GCTCATCTTCTCGGTGTCGTCCATG
**aHb5a**	PmaGb2for	ATGCCTATCGTTGACACTGGA
	PmaGb2rev	TTAGTAGGCGGACCTGAGC
	PmaGb08qPCRfor	CCGGTGTGGACATCCTGGTGAAGTTCT
	PmaGb08qPCRrev	ACGTCCGCGGATTTCTTGAGCTGA
**aHb6**	Pma_aHb6_for	TGAGCAGTTTCAAGGAGGATGAA
	Pma_aHb6_rev	TCTTGGTCGAAGCTGCTGA
	Pma_aHb6_qpcr_f	AGCTGTGCTCGACTTGGACGATC
	Pma_aHb6_qpcr_r	CCCGCCTACTTCAAGACGTTTGCAGA
**aHb7**	Pma_aHb7_for	TCTCGGACGAGGAGAAGAAG
	Pma_aHb7_rev	ATCTGCATCAGCCTCAAAGT
	Pma_aHb7_qpcr_f	CCGAAAAGAACGCCAAGAATCTGAAGGAGC
	Pma_aHb7_qpcr_r	CAAGTTCTTCAAGGTCCTGGCTGAGC
**aHb11**	Pma_aHb11_for	ATGCCTATTGTCGACTCGGGAAGC
	Pma_aHb11_rev	GCATCCTGCTGAAGTCCCAGTACTAG
	Pma_aHb11_qpcr_f	CCCGAGAAGCAGAGCCTGAAGC
	Pma_aHb11_qpcr_r	GACCCCCAGTATTTCAAGGTGCTGTCTG
**aHb12**	Pma_aHb14_for	GACTCTGGAAGCGTTGGG
	Pma_aHb14_rev	GGTGGTGATCCTGCTCAAG
	Pma_aHb12N_qpcr_f	GCCTTAGACGACCCCCCGAAG
	Pma_aHb12N_qpcr_r	TACTTCAAGGTTCTGGCTGGAGTCATTTCCG
**aMb1**	PmaGb17_for	ATGAGCATTGCAGACAGC
	PmaGb17_rev	GAATGCAGATTTCAAGCCAATGC
	PmaGb17_qPCR_for	CCACGCAGAGGACCACGGAACC
	PmaGb17_qPCR_rev	CAGCGCCTGGAACTTGGGGAAG
**aMb2**	PmaGb23_for	ATGAGTGCCATCGTGGAC
	PmaGb23_rev	GCCTAGAAAGCCGAGCG
	PmaGb23_qPCR_for	GCCAAGCTGCACTCGCTGAGC
	PmaGb23_qPCR_rev	GTCTCGCCCAGGATGTCGAGC
**Cygb**	PmaGb30for	ATGGAGCAGGGCTGGCTGT
	PmaGb30rev	CTAGGCCGCTGCTTGCTCC
	PmaGb30qPCRfor	CGTCAAGTACTACCACATCTTGGGCGG
	PmaGb30qPCRrev	AGGGTGCCCAGCAGCTTCGTC
