# Supplementary material for: Convergent evolution of hemoglobin switching in jawed and jawless vertebrates
Source: BMC Evol Biol. 2016 Feb 1;16:30. doi: 10.1186/s12862-016-0597-0 (PMC4736134; doi:10.1186/s12862-016-0597-0)
Supplement: Additional file 2: Figure A1. — Sequence alignment (FASTA format) of the globin sequences of P. marinus. (PDF 8 kb) [file 12862_2016_597_MOESM2_ESM.pdf]

Additional Fig. A1. Sequence alignment (FASTA format) of the globin sequences of *P. marinus.*

```
>aHb1
-MPIVDS------------------------------------GSVPALTAAEKATIRTA
WAPVYAKYQSTGVDILIKFFTSNPAAQAFFPKFQGLTSADQLKKSMDVRWHAERIINAVN
DAVVAMDDTEKMSLKLRELSGKHAKSFQ-VDPQYFKVLAAVIVDTVLP---------GDA
GLEKLMSMICILLRSSY---------------------------
>aHb2a
-MPIVDT------------------------------------GSVAPLSAAEKTKIRSA
WAPVYSNYETSGVDILVKFFTSTPAAQEFFPKFKGLTTADQLKKSADVRWHAERIINAVN
DAVVSMDDTEKMSMKLRDLSGKHAKSFQ-VDPQYFKVLAAVIADTVAA---------GDA
GFEKLMSMICILLRSAY---------------------------
>aHb2b
-MPIVDT------------------------------------GSVAPLSAAEKTKIRSA
WAPVYSNYETSGVDILVKFFTSTPAAQEFFPKFKGLTTADQLKKSADVRWHAERIINAVN
DAVVSMDDTEKMSMKLRDLSGKHAKSFQ-VDPQYFKVLAAVIADTVAA---------GDA
GFEKLMSMICILLRSAY---------------------------
>aHb2c
-MPIVDT------------------------------------GSVAPLSAAEKTKIRSA
WAPVYSNYETSGVDILVKFFTSTPAAQEFFPKFKGLTTADQLKKSADVRWHAERIINAVN
DAVVSMDDTEKMSMKLRDLSGKHAKSFQ-VDPQYFKVLAAVIADTVAA---------GDA
GFEKLMSMICILLRSAY---------------------------
>aHb5a
-MPIVDT------------------------------------GSVAPLSAAEKTKIRSA
WAPVYSTYETSGVDILVKFFTSTPAAQEFFPKFKGLTTADQLKKSADVRWHAERIINAVN
DAVASMDDTEKMSMKLRDLSGKHAKSFQ-VDPQYFKVLAAVIADTVAA---------GDA
GFEKLMSMICILLRSAY---------------------------
>aHb5b
-MPIVDS------------------------------------GSVAPLSAAEKTKIRSA
WAPVYSTYETSGVDILVKFFTSNPAAQEFFPKFKGLTTADQLKKSADVRWHAERIINAVN
DAVASMDDTEKMSMKLRDLSGKHAKSFQ-VDPQYFKVLAAVIADTVAA---------GDA
GFEKLMSMICILLRSAY---------------------------
>aHb5d
-MPIVDS------------------------------------GSVAPLSAAEKTKIRSA
WAPVYSTYETSGVDILVKFFTSNPAAQEFFPKFKGLTTADQLKKSADVRWHAERIINAVN
DAVASMDDTEKMSMKLRDLSGKHAKSFQ-VDPQYFKVLAAVIADTVAA---------GDA
GFEKLMSMICILLRSAY---------------------------
>aHb8
-MPIVDS------------------------------------GSVAPLSAAEKTKIRSA
WAPVYSNYETSGVDILVKFFTSTPAAQEFFPKFKGMTTADQLKKSADVRWHAERIINAVN
DAVTSMDDTEKMSMKLRDLSGKHAKSFQ-VDPQYFKVLAAVIADTVAA---------GDA
GFEKLMSMICILLRSAY---------------------------
>aHb3
-MPIVDS------------------------------------GSVAPLSAAEKTKIRSA
WAPVYSNYETTGVDILVKFFTSTPAAQEFFPKFKGLTTADQLKKSADVRWHAERIINAVN
DAVVSMDDTEKMSMKLGDLSGKHAKSFQ-VDPQYFKVLAAVIADTVAA---------GDA
GFEKLMSMICILLRSAY---------------------------
>aHb9
-MPIVDS------------------------------------GSVGALSGAEKAAIADS
WKAVYSNYEEAGKAILIKFFTSNPGVQDFFPKFKGLDSADQLSKSAAVRWHAERIINAVN
DAVVALDDPEKLSLKLKALSKKHAQEFN-VDPQYFKVLAVNIVEGVSSANG-GLGAEAQA
AWEKFLSQVSILLKSQY---------------------------
>aHb11
-MPIVDS------------------------------------GSAGALSAAEKAIITDS
WKVVYADYEAAGKAILIKFFTSNPGVQDFFPKFKGLDSADQLSKSAAVRWHAERIINAVN
DAVVALDDPEKQSLKLKALSKKHAQEFN-VDPQYFKVLSANVLEQVAAANG-GLSAEAQG
AWEKLLSIISILLKSQY---------------------------
>aHb10
```

-MPIVDS------------------------------------GSVGALSASEKAAVAGS
WKAVYANYEAAGKAVLIKFFTSNPGVQDFFPKFKGLDSADKLSKSPAVRWHAERIINAVN
DSVVALDDPEKQSLKLKALSQKHAYEFH-VDSQYFKVLSATILEQVDDANG-GLSAEGKS
GWEKLLSSICIHLKSAY---------------------------
>aHb12
-MPIVDS------------------------------------GSVGEFSAAEKSLIVSA
WAPVYAKYEEAGVDILVKFFSDNPGVQDFFPKFKGLDSADQLKKSPAVRWHAERIINAVN
DAVVALDDPPKLSLKLKALSKKHAQELN-VDPQYFKVLAGVISDAVAK------SGDEKA
AVDKFLSQVVILLKFAY---------------------------
>aHb13
-MPIVDS------------------------------------GSVGAISAAEKSLIVSA
WAPVYAKYEEAGVDILVKFFAANPEAQAFFPKFKGLDSADQLKKSPAVRWHAERIINAVN
DAVVALDDPAKQSLQLKALSQKHAHELN-VDPSYFKVLAGVISDAVAK------SGDAKA
AVDKFLSQVVILLKSAY---------------------------
>aHb14
-MPIVDS------------------------------------GSVGAISAAEKSLIVSA
WAPVYAKYEEAGVDILVKFFAANPEAQAFFPKFKGLDSADKLKKSPAVRWHAERIINAVN
DAVVALDDPAKQSLQLKALSQKHAHELN-VDPSYFKVLAGVISDAVAK------SGDAKA
AVDKFLSQVVILLKSAY---------------------------
>aHb7
-MPIEDT------------------------------------GSKPDFSDEEKKAIKDS
WSGVYSEYESASSEILIKFFVDNPSAQDFFPKFKDLDSEEKLKNSTAVRWHAERIINAVN
DVIWLLDEPEKNAKNLKELSEKHAVQLN-VDAKFFKVLAELILDKVAEKSGDSFSDSARS
AWEKLLTYICISLKVAY---------------------------
>aMb1
-MSIADS------------------------------------GSAPALSGDEKSAVRDT
WKVVYPHAEDHGTTILIKFLTENADAKKFFPKFQALKTADEMKSSPVLRDHAKRIMNSIN
DMVVALDDTNAQNAQMNGLSKKHANDFK-VDPKYFKVISNVILSVIAEGLGAQFNDAAKN
GWSKLLTTTCIGLKSAF---------------------------
>aMb2
MSAIVDS------------------------------------GSAPALSGDEKAAIKST
WPSVFAKAEDVGAEMLSRFISSNADVKKYFPKFKDISSQAELKSSAKVRDHAKRIMAFIN
DLVDNIDNAGAQTAKLHSLSAEHAEKFK-VDPQYFKVISNVLLDILGETFGASFSGATRS
AWIKLLSIICIGLRSAF---------------------------
>aHb6
MGALQDS------------------------------------GIVSSFKEDEKAALRES
WDIFNNSHQDAGVKILARFIINNPEAKKFFPKFKGLNTAEELQNSAEVRIHGDKILAAVQ
QAVLDLDDPDKQKNKLKDLSKSHAQQFN-VEPAYFKTFAEVILKYVTETCGKSFTSEMRT
SWSKLLSLIIIELQSAY---------------------------
>Cygb
---MEQGWLSEEEIEALQDI
WEKVFKSAEDVGVILLVRLFTGHPASKQYFPMFKDLETADDLKASAKLRWHAGRVMGSLD
KAVRSLRKPEELIKILRAVGLSHARKATPVDVKYYHILGGIIMDVLLETFKDELSPTARS
AWTKLLGTLCTEFENAYREE----------------GVLEQAAA
>GbX1
MGCTVSTDERTGAQSSSQGQSQASRKQQQPEQQRAAGEGHQPPGPPQAPSESQRRLVRDS
WLALQCDIARVGVIMFVRLFETHPECKDVFYQFRDCEDLQKLKMNKQLQAHGLRVMSFIE
KSVARLEQECVLEQLIVEMGRKH-YKYN-ASPKYYSFVGIEFIATVQPFLQEKWTNEVED
AWQCLFRYIAAVMKRGYLEEEAASNGVNTANYDRGQGNHGATAM
